# General perception and self-practice of complementary and alternative medicine (CAM) among undergraduate pharmacy students of Bangladesh

**DOI:** 10.1186/s12906-017-1832-y

**Published:** 2017-06-14

**Authors:** Bijoy Laxmi Saha, Md. Omar Reza Seam, Md. Mainul Islam, Abhijit Das, Sayed Koushik Ahamed, Palash Karmakar, Md. Fokhrul Islam, Sukalyan Kumar Kundu

**Affiliations:** 1 Department of Pharmacy, Noakhali Science and Technology University, Noakhali, -3814 Bangladesh; 2 Department of Pharmacy, Comilla University, Kotbari, Comilla, 3506 Bangladesh; 3 Department of Pharmacy, Jahangirnagar University, Savar, Dhaka, -1342 Bangladesh

**Keywords:** CAM, Awareness, Pharmacy, University students, Bangladesh

## Abstract

**Background:**

Complementary and Alternative Medicine (CAM) is a combination of herbal medicine, traditional therapies, and mind-body intervention. This descriptive study was designed to assess the knowledge, attitudes, perception and self-use of CAM among Bangladeshi undergraduate pharmacy students. The study also evaluated their opinions about its integration into the pharmacy course curriculum.

**Methods:**

It was a cross-sectional, questionnaire-based study conducted on 250 pharmacy students of five reputed public universities of Bangladesh.

**Results:**

This study revealed that majority of the pharmacy students were using or had previously used at least one type of CAM. Among the students, 59% had used homeopathy followed by Ayurveda (30%), meditation (29%), massage (13%), Unani (9%), yoga (6%) and acupuncture (2%). Students’ attitudes towards CAM were influenced by family and friends, books and journals, the internet and to a lesser degree by health practitioners. A significant (*p* < 0.05) number of students had knowledge about CAM. A majority of the students (90%) had positive, while 10% had negative attitudes towards CAM. Lack of knowledge and trained professionals were found to be the major interruptions to CAM use. 84.45% acknowledged the importance of knowledge about CAM for them as future healthcare practitioners. Furthermore, the majority of the students also believed that ideas and methods of CAM would be beneficial for conventional medicine.

**Conclusions:**

From the findings of the study, it can be recommended that an approach should be taken to educate the students about the fundamentals of CAM use so that it may fulfill the professional needs of our future pharmacists.

## Background

CAM has been defined by the National Center for Complementary and Alternative Medicine as a group of diverse medical and health care systems, practices, and products that are not presently considered to be part of conventional medicine [1]. They may be grouped into categories such as natural products, mind–body, and body-based practices. CAM is a broad domain of healing resource to improve the quality of life and can be a valuable addition to the chronic pain management plan [2]. CAM can be differentiated from traditional medicines (TM) as such traditional practices are based on a holistic approach to the mankind within the wider environment; it is a framework that reaches far beyond the field of health to the broader level of society, religion, and culture. The use of CAM is increasing rapidly in developed and developing countries whereas the use of TM remains comprehensive only in the developing countries [3].

The growth of conventional medicine has started at the beginning of nineteenth century but today almost 67% of the U.S. population report using at least one form of CAM [4]. In Bangladesh, CAM has long been practised and it is estimated that about 70–75% population of the country are still using traditional medicine for the management of their various health problems. Broadly speaking, four types of CAM are being primarily practised in Bangladesh namely herbal, homeopathy, religious and magical methods. Both registered and unregistered (locally known as kabiraj) herbal practitioners are practicing CAM in the country at present [5]. The herbal method of complementary and alternative medicine mainly consists of ayurvedic and unani systems in Bangladesh. Based on the existing rich local plant diversity, the tradition of indigenous herbal medicine systems has formed a very important component of the primary healthcare system of Bangladesh [6].

As a developing country the demand of complementary and alternative medicine is increasing day by day. Besides the public demand for more information regarding CAM, the understanding, perceptions and self-use of CAM among undergraduate health science students have become a topic of interest. This creates a challenge for the future pharmacists in order to gain adequate knowledge to recommend and counsel on CAM. Deeper understanding and acceptable perceptions about CAM among pharmacy students will be fundamental in developing a professional image as providers and advisors on conventional medicines and CAM [7, 8]. In 1999–2000, 28.7% of the rural population of Bangladesh used traditional complementary and alternative medicine (TCAM) for treatment or prevention of disease. Among them, 26% preferred Unani medicines, while 23% preferred Ayurveda. Again 37% of them used to visit practitioner, whereas 31% was initially self-medicated. They used TCAM for simple complications like fever (1.9% of respondents surveyed), cough and cold (7.1%), high blood pressure (2.5%), stomach pain (3.4%) and dysentery (1.6%) [5, 7].

Most of the TCAM practitioners were in the age group of 21–40 years (56%). TCAM was mainly used by people with education level below secondary school level (66%). Less than 1.4% of graduates and postgraduates seemed to prefer TCAM. It may be due to the lack of scientific evidence. Here 67% of the total users of TCAM belong to low-income population groups (up to 4000taka, per month) [5, 7]. As CAM is cost effective in Bangladesh, it is more preferred by low income professional groups.

Considering all the evidence, the aim of the study was to assess the awareness about CAM among the pharmacy students of Bangladesh. The study also focused on the knowledge level, perception and attitude of the students towards CAM and tendency of self-practices of CAM among them. We also collected information about different CAM therapies and barriers of CAM use. Finally the study also correlated the data with respondents’ yearlong study.

## Methods

### Study design and sampling

For this population-based cross-sectional study, respondent was selected from pharmacy department of five public universities namely Dhaka, Jhangirnagar, Chittagong, Noakhali Science and Technology and Comilla University between January to March 2016. A simple random sampling technique was used for the selection of study participants. From the equation used for sampling the number of respondents of this study was 233 but for the sake of proper result and data analysis data were collected from 238 undergraduate pharmacy students. Among them, 134 of them were male, and 104 were female. All the participants willingly joined in this study providing written consent.The study protocol was approved by the ethical research committee of the department of pharmacy of Noakhali Science and Technology University.

### Study questionnaire

The questionnaire was adopted from a formerly published study^7–10^ standardized for undergraduate pharmacy students. The questionnaire was divided into six portions: A, demographic information; B, knowledge of CAM; C, self-practice and use of CAM [9]; C, source of information about CAM [9, 10]; D, barriers of CAM use [8, 9, 11]; E, awareness and perceptions about CAM [9–11]; F, integration of CAM into curriculum [9].

### Data collection

The procedure of data collection was segmented into three steps. The first step was to fill up the questionnaire including socio-demographic and anthropometric information by the study subjects. The second phase was to focus on group discussion about the study protocol, and the final step was to cross-check interviews with the key informants. The questionnaires were distributed among the selected students together with a written consent form that explained the purpose of the research and assured them of their confidentiality. The respondents were given an explanation of the therapy, its most important principles and evidence base and a practical demonstration for about fifteen minutes. They were invited to complete the questionnaire immediately. The authors were present on hand to answer questions or clarify any doubt that they might have. The class leader collected all the filled up questionnaires in each class.

### Statistical analysis

All data obtained were entered into a Microsoft Excel spreadsheet and exported for analyses using SPSS software version 16.0 (SPSS Inc., Chicago, IL., USA). Descriptive statistics were used for the calculation of proportions. Chi-square test was performed to measure the association between the demographic characteristics and responses to understanding, perceptions, and self-use of CAM. The *p* values were calculated by chi-square and Spearmen correlation test. An alpha level of 0.05 or less was considered significant. Microsoft Excel program was used for data analysis and chart, graph, and diagram preparation.

## Results

### Demographic characteristics

The Table 1 showed that majority of the students were male with a percentage of 56% while female students comprised of 44%. Among all pharmacy students, 26% were from 1st year, 27% from the 2nd year, 26% from the 3rd year, and 20% from the 4th year. Lowest number of students were in age group <18 years with a percentage of 1% while the highest number of students were in the 21–25 years age group with a rate of 65%. Another 34% students were in the age group 18–20 years. Among the respondents 82% respondents were from the urban area and 19% were from the rural area.

**Table 1:** Demographic characteristics of respondents

Item	Subgroup	Number	Percentage (%)
Sex	Male	134	56
	Female	104	44
Year of study	1st Year	62	26
	2nd year	65	27
	3rd year	63	27
	4th year	48	20
Age group	<18 y	2	1
	18–20 y	82	35
	21–25 y	154	65
Area of residence	Urban	195	82
	Rural	43	18

### Self-practice or use of CAM

Table 2 describes the habit of using CAM from 1st year to 4th-year pharmacy students of five reputed public universities. Among them average 90% student had the experience of using CAM while 10% students never used CAM before.

**Table 2:** Self-practice or use of CAM

Item	Response	Year of study (%)				Total (%)	*p*-value
		1st	2nd	3rd	4th		
Use of CAM	Yes	87	92	92	90	90	0.49
	No	13	8	8	10	10	
Type of CAM used							
Meditation	Yes	27	38	32	15	29	0.167
	No	73	62	68	85	71	
Spirituality	Yes	11	3	0	0	4	0.017^*#^
	No	89	97	100	100	96	
Homeopathy	Yes	56	55	65	60	59	0.678
	No	44	45	35	40	41	
Ayurveda	Yes	27	32	30	31	30	0.943
	No	73	68	70	69	70	
Unani	Yes	11	12	5	8	9	0.459
	No	89	88	95	92	91	
Acupuncture	Yes	3	2	2	2	2	0.905
	No	97	98	98	98	98	
Massage	Yes	21	12	11	4	13	0.067
	No	79	88	89	96	83	
Hypnosis	Yes	2	0	2	2	2	0.949^#^
	No	98	0	98	98	98	
Yoga	Yes	6	8	8	0	6	0.504^#^
	No	94	92	92	100	94	
Other		3	0	0	2	1	-
Purpose of using CAM							
Severe disease	Yes	34	18	32	8	24	0.005^*^
	No	66	82	69	92	76	
Common cold & flu	Yes	23	46	33	38	35	0.046^*^
	No	77	54	68	62	65	
Pain & inflammation	Yes	34	26	43	27	33	0.176
	No	66	74	57	73	67	
Cost effectiveness	Yes	10	12	21	21	16	0.220
	No	90	88	79	79	84	
Easy availability	Yes	24	28	27	17	24	0.538
	No	76	72	73	83	76	
Other		3	2	0	17	5	-
Effectiveness of result	Yes	92	98	90	85	92	0.081
	No	8	2	10	15	8	
Recommending CAM	Yes	92	94	90	77	90	0.026^*^
	No	8	6	10	23	10	
Adverse effect	Yes	18	12	11	10	13	0.628
	No	82	88	89	90	87	

*P < 0.05 was considered as significant; ^#^for zero cells Yates correction has been conducted

From the table, we see that use of spirituality for the treatment differed significantly (*p* < 0.05) when compared to those who did not use these therapies for treatment and the total percentage of using these therapy was 4%. As spirituality has no scientific evidence therefore the 3rd and 4th year students did not use spirituality as they had better knowledge than the 1st and 2nd year students. Meanwhile, the average rate of using homeopathy was highest (59%) whereas Ayurveda, meditation, massage, Unani, Yoga, acupuncture, comprised of 30%, 29%, 13%, 9%, 6%, 2% respectively. It was found that among different therapies, students used hypnosis at least with an average percentage of 1%.

The purpose of using CAM by students was categorized into five broad categories. Majority of the students used CAM therapy in case of common cold and flu (35%). Pain and inflammation were the second reason for using CAM with a percentage of 33%. On the other hand 24% students’ used CAM due to easy availability, 24% used it in the case of some severe diseases, and 16% used it in the event of cost effectiveness. The result was significant (*p* < 0.05) only in the event of treatment of severe diseases.

Among the students, 92% students got the useful result using CAM therapy, and for 8% the result was negative. It was found from the result that 90% students were keen to recommend CAM to others while 10% were not interested in doing so. 13% students experienced adverse effect after using CAM, but the majority (87%) had not reported any adverse effect of CAM.

### Sources of information about CAM

**Fig. 1:**
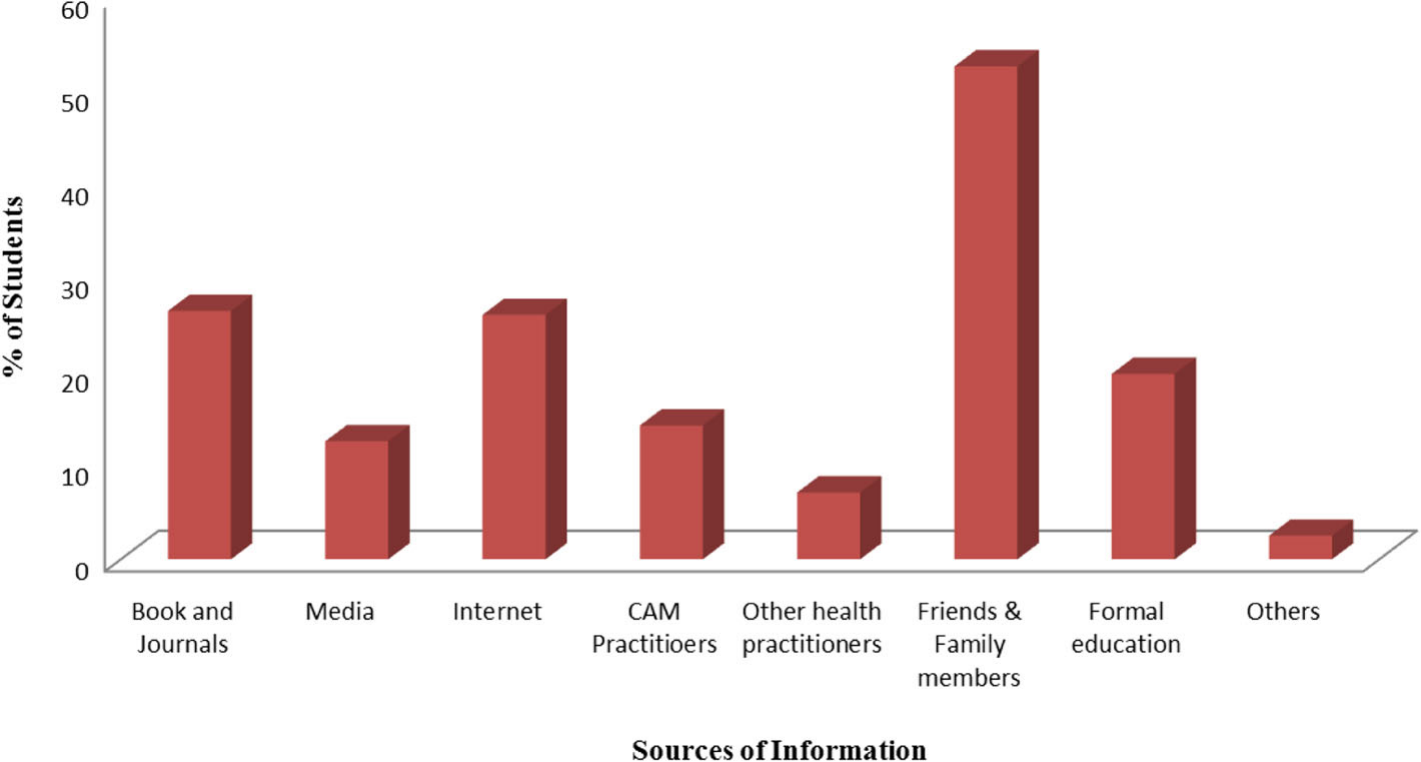
Sources of information about CAM among students

Figure 1 describes the sources of information about CAM acquired by pharmacy students of five universities. The diagram showed that majority of the students (53%) of different educational years considered friends and family as the primary source of information about CAM. Whereas the second source of information about CAM was the books and journals (26%) and internet (26%). About 20% students came to know about CAM from formal education while another 14% and 7% student knew about CAM from CAM practitioners and other health practitioners respectively. Media was the source of CAM information for about 12% students.

### The barrier of CAM study

**Fig. 2:**
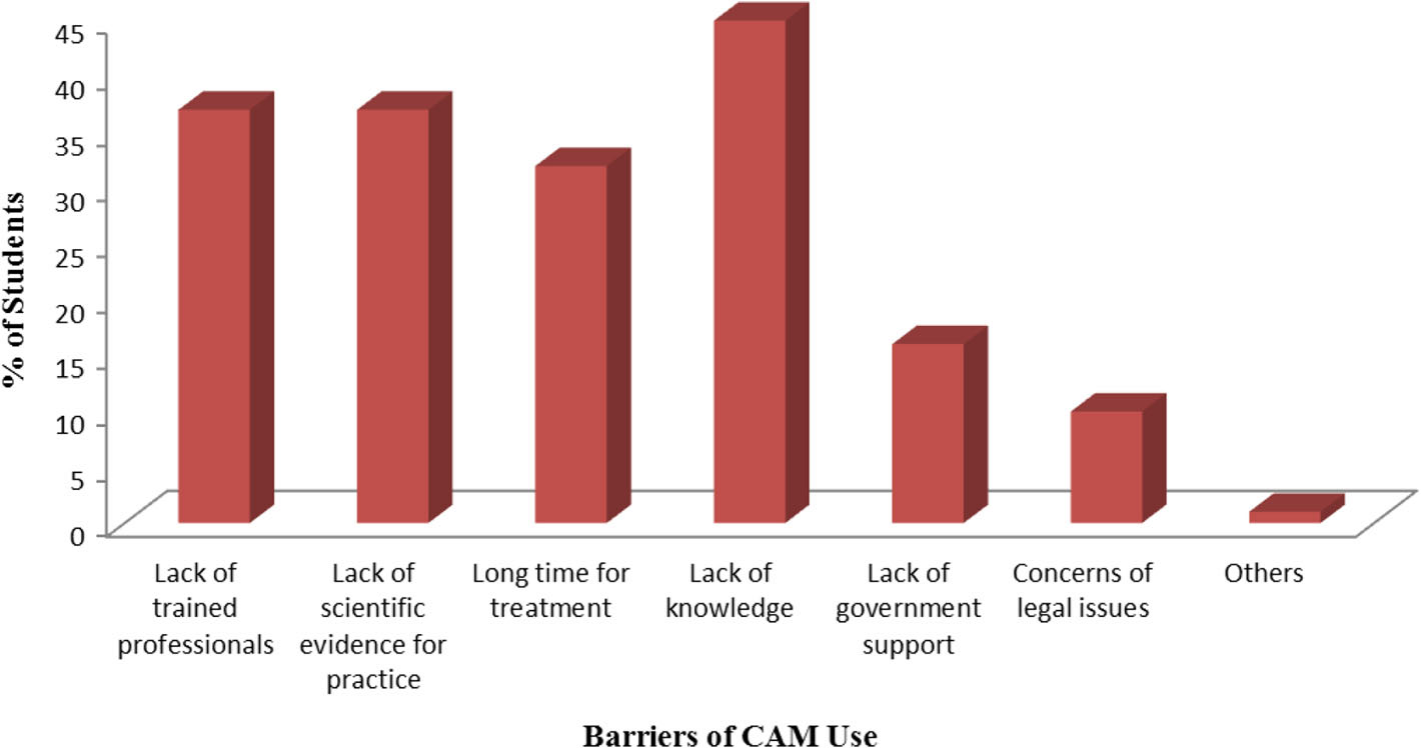
Barrier of CAM use

Figure 2 represents the 1st year, 2nd year, 3rd year and 4th year pharmacy students’ opinion about the restriction of CAM use. Almost 45% students considered the lack of knowledge as the prime obstacle to CAM use. Lack of scientific evidence and lack of trained professionals were considered as the barrier of CAM use by 37% students. Taking long time for treatment was marked as the 3rd major barrier of CAM use by the students (32%). Meanwhile, lack of government support and concerns of legal issue were mentioned as the obstacles to CAM practice by another 16% and 10% of the students respectively.

### Awareness and perception about CAM

Table 3 represents the students’ knowledge and understanding about CAM by analyzing their answer level given in “yes” and “no”. Greater percentage of students had a positive attitude about use and benefit of CAM while a very negligible amount of students had a negative attitude about it.

**Table 3:** Awareness and perception about CAM

Modality	Year of study	CAM use (Yes/no)	Total (%) (Yes/no)	Chi-square value (χ^2^)	*P* value
Knowledge of CAM	1st	49/13	88/12	12.710	0.005^*^
	2nd	55/10			
	3rd	62/1			
	4th	44/4			
Health benefit	1st	61/1	97/3	5.549	0.136
	2nd	64/1			
	3rd	58/5			
	4th	47/1			
Right to choose	1st	61/1	97/3	1.076	0.783
	2nd	63/2			
	3rd	60/3			
	4th	46/2			
CAM together with conventional medicine	1st	55/7	82/18	4.627	0.201
	2nd	52/13			
	3rd	52/11			
	4th	35/13			
Quackery and charlatanism	1st	2/60	5/95	1.748	0.626
	2nd	4/61			
	3rd	4/59			
	4th	1/47			
Discouraging unscientific manner	1st	51/11	80/20	0.462	0.927
	2nd	52/13			
	3rd	50/13			
	4th	37/11			
Placebo effect	1st	14/48	18/82	2.310	0.511
	2nd	9/56			
	3rd	13/50			
	4th	7/41			
Stimulant of body’s natural power	1st	34/28	66/34	6.022	0.111
	2nd	43/22			
	3rd	42/21			
	4th	37/11			
Ideas & methods of CAM for convention medicine	1st	57/5	94/6	6.116	0.106
	2nd	65/0			
	3rd	57/6			
	4th	45/3			
Advising CAM by health professional	1st	61/1	93/7	4.748	0.191
	2nd	61/4			
	3rd	56/7			
	4th	44/4			
Threat to public health	1st	7/55	11/89	2.405	0.493
	2nd	6/59			
	3rd	5/58			
	4th	8/40			

*P < 0.05 was considered as significant

It can be seen from the Table 3 among the students about 885 had significant knowledge (χ^2^ = 12.710; *p* < 0.05) about CAM use. About 97% of the students said that they knew that CAM use is beneficial for health and they have the right to choose between CAM and conventional medicines. When asking about use of CAM together with conventional medicine 82% student opined that they were using CAM together with conventional medicine (χ^2^ = 4.627; *p* = 0.201). Majority (95%) of the students don’t think that CAM is quackery and charlatanism (χ2 = 1.748; *p* = 0.626) but 80% of them believe that treatments not tested in a scientifically recognized manner should be discouraged (χ^2^ = 0.462; *p* = 0.927). 82% students figured out that CAM therapies are not the results of a placebo effect (χ^2^ = 2.310; *p* = 0.511) while 66% students think that CAM therapies can stimulate the body’s natural therapeutic powers. However, 94% students considered that it would be beneficial for conventional medicine if some ideas and methods of CAM therapies can be integrated with it (χ^2^ = 6.116; *p* = 0.106). The study results also highlighted that about 93% students (χ^2^ = 4.748; *p* = 0.191) think that health professionals should advice or encourage patients to use some of the common CAM therapies where majority of the students (89%) pointed out that CAM therapies are not a threat to public health (χ^2^ = 2.405; *p* = 0.493).

### Integration of CAM into the curriculum

**Fig. 3:**
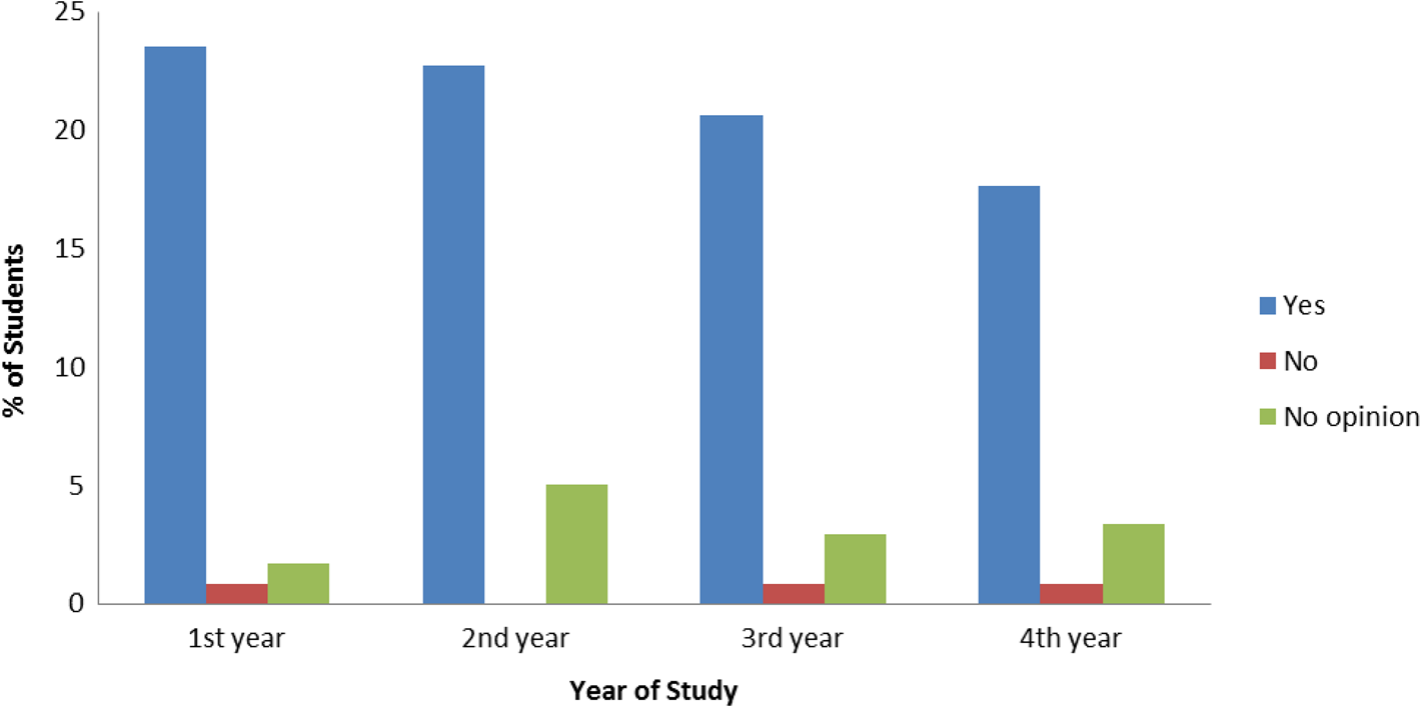
Knowledge of CAM for pharmacist and health professional

Figure 3 showed students’ opinion about the necessity of knowledge about CAM for pharmacists and health professionals. Most of the students from different years were confident about the need for knowledge of CAM for pharmacists and health professionals and among them, the majority were the 1st year students. Very few students from 1st year, 3rd year and 4th year disagreed with this.

**Fig. 4:**
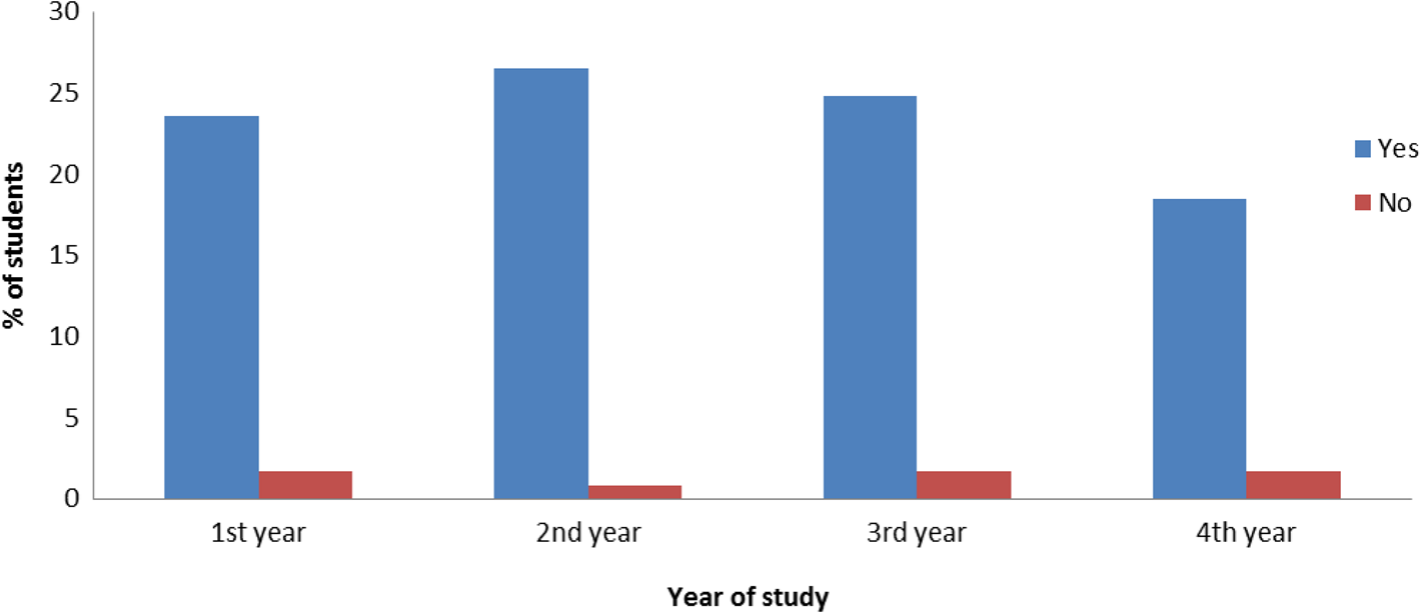
Investigation of CAM in universities

Figure 4 deals with the necessity of investigation of CAM in universities. Almost all of the students from all study year of all universities agreed with the need for research of CAM in universities. Very few students disagreed with it.

## Discussion

The observed rate of CAM usage by the respondents found in this study is very high (90.34%) which is near to the result found in the study on Australian pharmacy students [10]. Almost all of the respondents have used at least one type of complementary and alternative therapy. More than half (59.24%) of the students have the experience of using homeopathy while very few (1.26%) students have experience of using hypnosis. Use of homeopathy medicine by Bangladeshi students is very high compared to the pharmacy and medical students in Turkey [12], Sierra Leone [9], and Malaysia [8]. But it is near to the study conducted in India [13] and Pakistan [14]. Popularity of this homeopathy medicine may be due to its easy availability and acquaintance. Majority of the respondents use CAM therapy in case of common cold and flu (34.87%) and for pain and inflammation (32.77%) which is significantly higher than the result found in a sample survey conducted in 1999–2000 in rural areas of Bangladesh where 1.9% respondents used CAM for fever and 7.1% respondents used CAM for cough and cold. [7]. Most of the users of CAM have no experience of adverse effect and it is beneficial to almost 92% students which is near to the result found on adult users in Enugu urban, Southeast Nigeria [15]. In our study group, friends and family, book journals and internet were the main information sources for CAM. According to a previous study conducted on Ghana medical students, Television was the main source of information on most of the CAMs, with family relations, friends and schools which provided knowledge especially in relation to Herbal medicine and traditional African medicine [16].

Lack of knowledge, lack of trained professionals, and lack of scientific evidence for practice are considered as the major impediments to CAM use to Bangladeshi public university students. Similarly in another study in Sierra Leone, lack of trained professionals, lack of knowledge of CAM and scientific evidence for practice were considered as the most common checks to CAM practice [9].

Regarding the awareness and use of the CAM modalities this study revealed that majority of the pharmacy students are conscious. First year students have comparatively less knowledge about CAM. Majority of the students have positive attitude towards CAM practice. In case of right to choose option between CAM and conventional medicine almost all students agreed with CAM. There was no significant difference in knowledge of CAM modalities with respect to gender and locality where a person grew up. Positive general attitude is reported among more than 85% medical students in Singapore [11]. In some studies female medical students were found to have more positive attitude towards CAM than male students [17, 18].

The study informs the calls for inclusion of CAM course at the undergraduate level of pharmacy; a move widely supported by nearly all of the pharmacy students (84.45%) irrespective of gender, age, level of study and religion. Although the student preferences were with regards to the mode of learning, as well as which academic year CAM should be introduced in. This was almost similar to the figures reported in the Western literature where it varied from 72% to 91% [11]. The high degree of receptivity for CAM suggests the need for curriculum development in this field. Medical and pharmacy schools in the West are increasingly introducing CAM education in the curriculum [19].

### Limitations

The study had some limitations as we faced some complications during the survey. Firstly, we had covered only five universities due to the shortage of time for the research work. So the represented data does not give the whole scenario of all the pharmacy students of the country. Secondly, students of 1st year and 2nd year were less familiar with some terminologies and complications arose regarding understanding the questionnaire. They needed further explanation. Thirdly, there was shortage of time as it was a project work. Fourthly, all universities were far away due to which it was a little bit difficult to travel these long distances.

## Conclusion

In Bangladesh, most of the undergraduate students use CAM medicine for the treatment of some simple complications like pain, inflammation, cold and flu. The use of CAM is considered to be effective and safe although their perception is not effected by their year of study. Men are being resistant to conventional medicine day by day. In this situation necessity of CAM has become crucial. To increase the CAM practice, knowledge and safety evidence about CAM is essential. CAM can be a valuable addition to the future treatment strategy in primary care and pharmacy students should learn deeply with evidence supporting their use and also showing their limitations. As CAM covers a broad range of diagnostic and therapeutic methods which include many medical fields, it seems to be important to combine general information on the topic with accurate information in the various therapeutic situations. It is encouraging that majority of the students are keen to integration of CAM into pharmacy course curriculum which will go a long way for enabling CAMs to contribute towards the overall health needs of Bangladesh.

## Abbreviations

CAM: Complementary and alternative medicine
NCCAM: National center for complementary and alternative medicine
SPSS: Statistical Package for the Social Sciences
TCAM: Traditional complementary and alternative medicine
